# Supplementary material for: Causal Relationship between Aging and Anorexia Nervosa: A White-Matter-Microstructure-Mediated Mendelian Randomization Analysis
Source: Biomedicines. 2024 Aug 16;12(8):1874. doi: 10.3390/biomedicines12081874 (PMC11351342; doi:10.3390/biomedicines12081874)
Supplement: Supplementary file 1 [file biomedicines-12-01874-s001.zip › Supplementary Figure/Figure S4/Singe SNP plot of MR analysis for TL to ubm-b-1758.pdf]

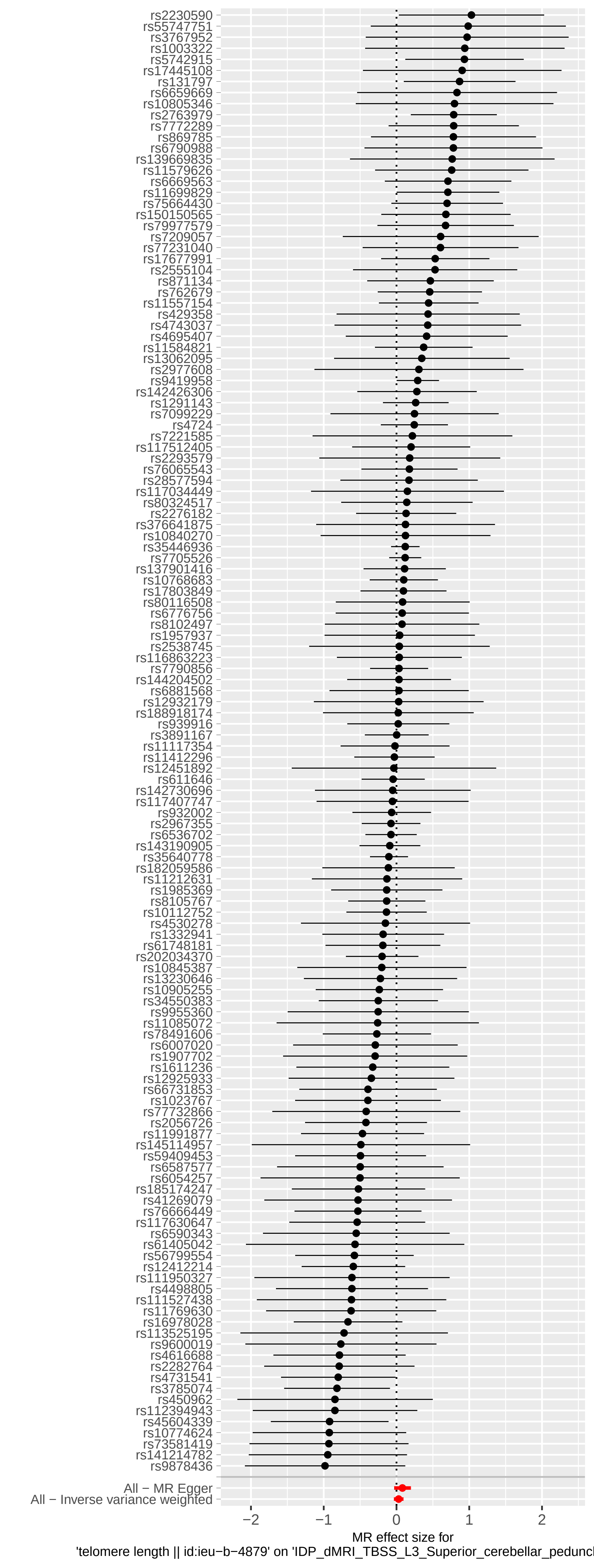

rs2230590
rs55747751
rs3767952
rs1003322
rs5742915
rs17445108
rs131797
rs6659669
rs10805346
rs2763979
rs7772289
rs869785
rs6790988
rs139669835
rs11579626
rs6669563
rs11699829
rs75664430
rs150150565
rs79977579
rs7209057
rs77231040
rs17677991
rs2555104
rs871134
rs762679
rs11557154
rs429358
rs4743037
rs4695407
rs11584821
rs13062095
rs2977608
rs9419958
rs142426306
rs1291143
rs7099229
rs4724
rs7221585
rs117512405
rs2293579
rs76065543
rs28577594
rs117034449
rs80324517
rs2276182
rs376641875
rs10840270
rs35446936
rs7705526
rs137901416
rs10768683
rs17803849
rs80116508
rs6776756
rs8102497
rs1957937
rs2538745
rs116863223
rs7790856
rs144204502
rs6881568
rs12932179
rs188918174
rs939916
rs3891167
rs11117354
rs11412296
rs12451892
rs611646
rs142730696
rs117407747
rs932002
rs2967355
rs6536702
rs143190905
rs35640778
rs182059586
rs11212631
rs1985369
rs8105767
rs10112752
rs4530278
rs1332941
rs61748181
rs202034370
rs10845387
rs13230646
rs10905255
rs34550383
rs9955360
rs11085072
rs78491606
rs6007020
rs1907702
rs1611236
rs12925933
rs66731853
rs1023767
rs77732866
rs2056726
rs11991877
rs145114957
rs59409453
rs6587577
rs6054257
rs185174247
rs41269079
rs76666449
rs117630647
rs6590343
rs61405042
rs56799554
rs12412214
rs111950327
rs4498805
rs111527438
rs11769630
rs16978028
rs113525195
rs9600019
rs4616688
rs2282764
rs4731541
rs3785074
rs450962
rs112394943
rs45604339
rs10774624
rs73581419
rs141214782
rs9878436
All − MR Egger
All − Inverse variance weighted
−2
−1
0
1
2
MR effect size for
'telomere length || id:ieu−b−4879' on 'IDP_dMRI_TBSS_L3_Superior_cerebellar_peduncl
